# Construction of a plasmid coding for green fluorescent protein tagged cathepsin L and data on expression in colorectal carcinoma cells

**DOI:** 10.1016/j.dib.2015.09.022

**Published:** 2015-09-30

**Authors:** Tripti Tamhane, Brit K. Wolters, Rukshala Illukkumbura, Gunhild M. Maelandsmo, Mads H. Haugen, Klaudia Brix

**Affiliations:** a Department of Life Sciences and Chemistry, Jacobs University Bremen, Campus Ring 1, D-28759 Bremen, Germany; b Department of Tumor Biology, Institute for Cancer Research, Oslo University Hospital – The Norwegian Radium Hospital, Oslo, Norway

**Keywords:** Cysteine cathepsin L, Green fluorescent protein, Colorectal carcinoma

## Abstract

The endo-lysosomal cysteine cathepsin L has recently been shown to have moonlighting activities in that its unexpected nuclear localization in colorectal carcinoma cells is involved in cell cycle progression (Tamhane et al., 2015) [1]. Here, we show data on the construction and sequence of a plasmid coding for human cathepsin L tagged with an enhanced green fluorescent protein (*p*hCL-EGFP) in which the fluorescent protein is covalently attached to the C-terminus of the protease. The plasmid was used for transfection of HCT116 colorectal carcinoma cells, while data from non-transfected and *p*EGFP-N1-transfected cells is also shown. Immunoblotting data of lysates from non-transfected controls and HCT116 cells transfected with *p*EGFP-N1 and *p*hCL-EGFP, showed stable expression of cathepsin L-enhanced green fluorescent protein chimeras, while endogenous cathepsin L protein amounts exceed those of hCL-EGFP chimeras. An effect of *p*hCL-EGFP expression on proliferation and metabolic states of HCT116 cells at 24 h post-transfection was observed.

**Specifications Table**

**Table:** 

Subject area	Molecular cell biology
More specific subject area	Cathepsin L protease mis-trafficking to the nucleus of colorectal cancer cells
Type of data	Plasmid sequence data, vector map, graph, figure
How data was acquired	Isolation of cathepsin L mRNA from HaCaT cells, reverse transcription to cDNA and vector construction by insertion into *p*EGFP-N1 (Clontech) using restriction sites *NheI* and *BamHI*, transfection of HCT116 colorectal carcinoma cells (ATCC) through X-treme GENE HP DNA transfection reagent (Roche Diagnostics), quantitation of cell proliferation rates and metabolic activity by MTT conversion assays, SDS-PAGE of whole cell lysates and immunoblotting with anti-cathepsin L antibodies and chemi-luminescence detection
Data format	Analyzed
Experimental factors	Cells transfected with two different plasmids were used in comparison with non-transfected controls
Experimental features	Proliferation and metabolic activity of transfected and non-transfected cells were quantified, and the molecular mass of expressed hCL-EGFP protein was determined in whole cell lysates by SDS-PAGE and immunoblotting.
Data source location	Bremen, Germany
Data accessibility	Data is provided in the article

**Value of the data**

• This article provides information on the construction of a vector coding for human cathepsin L-EGFP chimeras, and it describes the insert׳s sequence.
• The data provide detailed information on the molecular forms of cathepsin L chimeras with enhanced green fluorescent protein that are expressed in the human colorectal carcinoma HCT116 cell line.
• The article provides data on the effects of cathepsin L-EGFP expression in HCT116 cells with regard to proliferation rates and metabolic activity.

**Data**

EGFP-tagging is used to visualize transport and trafficking of cellular proteins including cysteine cathepsin proteases [2]. We have shown that EGFP-tagging does not interfere with intrinsic sorting signals at the N-terminus of cysteine cathepsin proteases when the tag is attached to the C-terminus via a spacer peptide [2–4]. In addition, transport to endo-lysosomal compartments and other cellular destinations is not affected by the EGFP tag [2–4]. Likewise, proteolytic activity is pertained in cathepsin-EGFP chimeras expressed in mammalian cell lines [2–7].

We have shown that cathepsin L is sorted and mis-trafficked to the nucleus of HCT116 cells as full-length protein and in proteolytically active form [1]. More specifically, nuclear cathepsin L accelerates cell cycle progression of HCT116 cells which are hyper-proliferative colorectal carcinoma cells. In this article, we demonstrate via expression of cathepsin L-EGFP chimeras the effects of enhanced protease expression promoting proliferation of HCT116 cells.

## 1 Experimental design, materials and methods

In this article, we describe the construction of a vector encoding human cathepsin L-EGFP (hCL-EGFP) with cDNA derived from cathepsin L mRNA of human HaCaT keratinocytes upon insertion into the multiple cloning site of *p*EGFP-N1 by *NheI* and *BamHI* restriction. Here, we share the relevant sequence data of *p*hCL-EGFP. The vector is introduced through lipofection to express chimeric, EGFP-tagged cathepsin L in HCT116 cells. This colorectal carcinoma cell line was further analyzed with regard to proliferation and metabolic activity by MTT-assays upon transfection with *p*hCL-EGFP in comparison to non-transfected and *p*EGFP-N1-transfected cells. The molecular forms of protein chimeras expressed in HCT116 cells were identified by immunoblotting of whole cell lysates separated by SDS-PAGE.

### 1.1 RNA preparation from HaCaT cells

The human keratinocyte line HaCaT (Human adult low Calcium high Temperature) was provided by Prof. Dr. Petra Boukamp (Deutsches Krebsforschungszentrum DKFZ, Heidelberg, Germany) and cultured in Dulbecco׳s modified Eagle׳s Medium (DMEM; Cambrex Corp., Verviers, Belgium) supplemented with 10% fetal calf serum (FCS; Gibco™ Invitrogen GmbH, Karlsruhe, Germany). Total RNA was isolated from phosphate-buffered saline (PBS)-washed confluent HaCaT cell cultures using TRIZOL reagent (TRIZOL; Invitrogen, Karlsruhe, Germany) with subsequent chloroform and isopropanol purification. The RNA pellet was washed with 75% ethanol and finally re-suspended in 30 µl of DEPC-treated water (Ambion, Huntigdon, UK). The RNA concentration was determined by nanodrop microphotometry (Kisker, Steinfurt, Germany) before aliquots were either directly used or snap-frozen in liquid nitrogen and stored at −80 °C.

## 1.2 Vector construction

Total RNA at a concentration of 0.05 µg/µL was reverse transcribed with a cDNA cycle kit (Invitrogen, Karlsruhe, Germany) using oligo dT primers and AMV reverse transcriptase. Amplification of the complete cathepsin L coding sequence was by PCR with 500 ng cDNA using 0.5 µM each of the primers “CL for *NheI*” (5′-aca cag gtt tta aaa cat gaa tcc tac a-3′) and “CL rev *BamHI*” (5′-agc tac ccc act gtg tga gct ggt gga-3′), 0.125 mM each of dNTPs, and 0.4 U Phusion DNA polymerase (Finnzymes, Espoo, Finland). By this cloning strategy, restriction sites for *NheI* and *BamHI* were introduced and the stop codon was removed, thus eventually yielding the sequence for a chimeric protein with the full-length cathepsin L sequence covalently connected to the EGFP tag by a spacer peptide linker. The final PCR product and *pEGFP*-N1 plasmid were each digested with *NheI* and *BamHI* (both, MBI, St. Leon-Rot, Germany). The restricted cDNA was ligated into the linearized vector using T4-DNA ligase (MBI) in the presence of ATP-containing reaction buffer (MBI) at 4 °C overnight, followed by ligase inactivation at 65 °C for 10 min. The ligated plasmids were transformed into competent *E. coli* JM 109, and kanamycin-resistant clones were used for isolation of vector cDNA (Qiagen, Hilden, Germany), which was eluted and stored in 10 mM Tris–HCl at pH 8.0. Control digests of the isolated plasmids were performed with *NheI* and *BamHI*, each used at 0.25 U/µg plasmid DNA, for insert restriction, and additionally *EcoRI* with a restriction within the insert was used (all, MBI) before analyzing on 1% agarose gels by electrophoresis. Finally, sequencing using the primers “CMV-Profor” (5′-aaa tgg gcg gta ggc gtg-3′) and “EGFP-Nrev” (5′-cgt cgc cgt cca gct c-3′) confirmed the correct product (Sequiserve, Vaterstetten, Germany).

## 1.3 Transfection of HCT116 cells

The human colorectal carcinoma HCT116 cell line was purchased from ATCC (Teddington, Middlesex, UK). The cells were cultured at 37 °C in a 5.0% CO_2_-atmosphere (Heraeus Instruments GmbH, Osterode, Germany) in RPMI-1640 medium (Biowhittaker™) supplemented with 10% fetal calf serum (Lonza, Verviers, Belgium). Transfection with *p*EGFP-N1 and *p*hCL-EGFP was performed using X-treme GENE HP DNA transfection reagent (Roche Diagnostics, Mannheim, Germany) following the manufacturer׳s protocol.

## 1.4 MTT assays

Cell proliferation rates and metabolic activity levels of transfected HCT116 cells were examined by a colorimetric assay in which the yellow 3-[4, 5-dimethylthiazol-2-yl]-2,5-diphenyltetrazolium bromide (MTT) is reduced to form purple formazan crystals by intracellular NAD(P)H-oxidoreductase. The assays were performed in duplicates and repeated at least twice as previously described [8], and the reaction product was quantified at 595 nm with a Tecan GENios Reader (Tecan Deutschland GmbH, Crailsheim, Germany).

## 1.5 Lysate preparation, SDS-PAGE, and immunoblotting

Transfected HCT116 cells were used 24 h post-transfection, and whole cell lysates were prepared in PBS containing 0.2% Triton X-100 as described before [9], normalized to equal amounts of protein and loaded onto 12.5% SDS-polyacrylamide gels (GE Healthcare, 80-1255-53) along with a PAGE ruler pre-stained protein ladder (Fermentas). After transfer onto nitrocellulose, blots were incubated with goat anti-human cathepsin L (1:500, Neuromics GT15049) and rabbit anti-human β-tubulin (1:1000, Abcam 6046-100) with HRP-coupled secondary antibodies used for subsequent visualization by enhanced chemi-luminescence onto CL-XPosure film (Pierce, through Perbio Science Europe, Bonn, Germany).

## 2 Results

Cathepsin L has been shown to be mis-trafficked in HCT116 cells and to enter the nucleus as an unexpected scene of potential action [1]. Nuclear cathepsin L is suggested to involve in regulation of cell cycle progression of HCT116 cells by accelerating S phase [1]. Moreover, the vector *p*hCL-EGFP coding for EGFP-tagged cathepsin L was expressed in HCT116 cells and sorted to the nucleus [1, Fig. 7D′].

### 2.1 Construction of the *p*hCL-EGFP vector

**Fig. 1:**
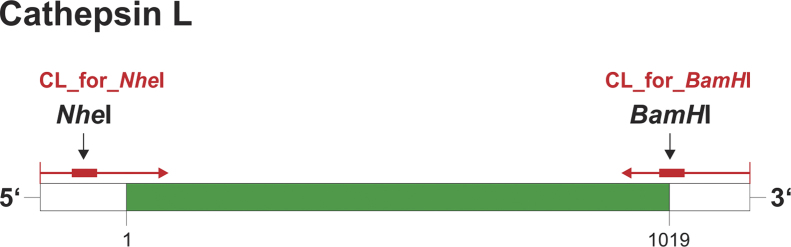
Scheme of specific amplification primers and expected amplicon. Forward and reverse primers for gene-specific amplification of cathepsin L are indicated in red and their amplicon is highlighted in green. Cleavage sites of *NheI* and *BamHI* are indicated by arrows. Numbers denote the length of the gene.

Vector construction was performed upon cloning of human cathepsin L from non-tumorigenic HaCaT keratinocytes and cDNA was inserted into the *p*EGFP-N1 vector (Clontech, Heidelberg, Germany), resulting in the fusion of sequences coding for cathepsin L and eGFP in *p*hCL-EGFP. The cloning strategy (Fig. 1) involved removal of the stop-codon at the 3′-end of cathepsin L cDNA, and introduced restriction sites for *NheI* and *BamHI* at the 5′- and 3′-ends, respectively, to ensure that the complete coding sequence was amplified. Removal of the stop-codon by *BamHI* digestion permitted further transcription once the gene was ligated in-frame into the suitable vector *p*EGFP-N1 to obtain mRNA coding for a fusion construct of cathepsin L and the marker protein enhanced GFP.

**Fig. 2:**
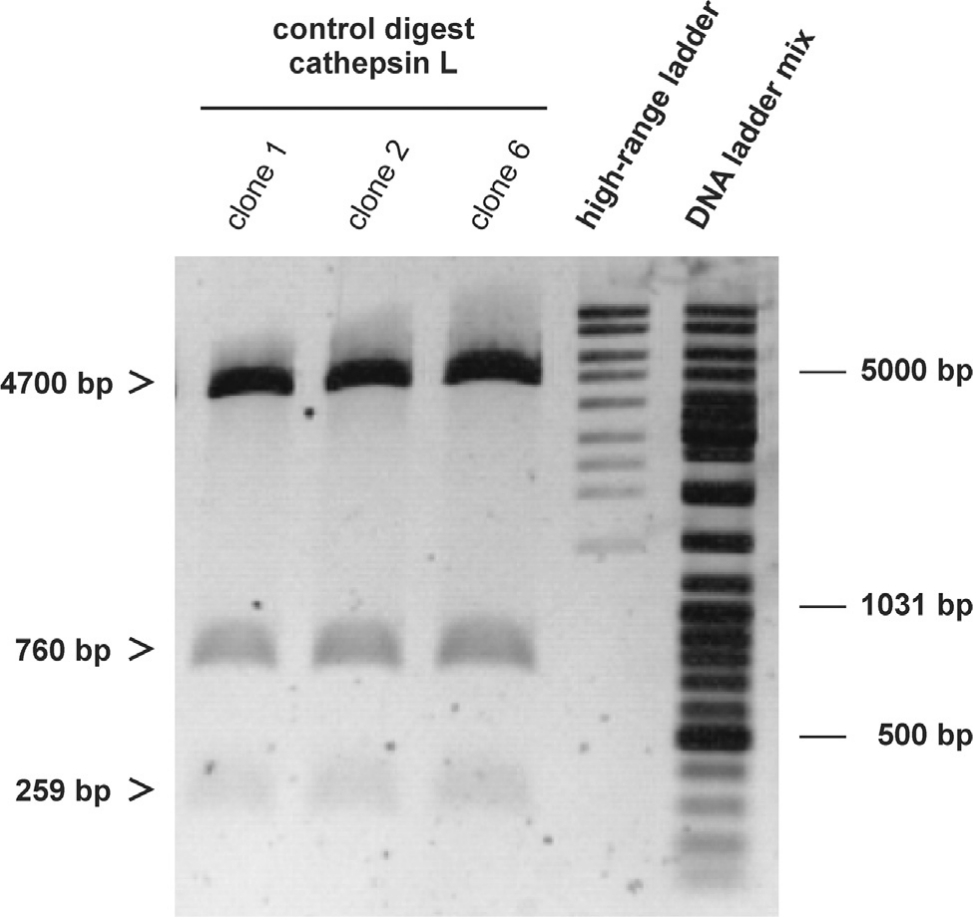
Agarose gel of control digest of *p*hCL-EGFP. Agarose gel of harvested plasmid *p*hCL-EGFP from three different clones after colony PCR and digestion with *NheI*, *BamHI*, and *EcoRI* is depicted. The expected fragments for cathepsin L-inserts and linearized vector with a length of 4700 bp, 760 bp, and 259 bp were detected after digest (arrowheads).

Transformed *E. coli* colonies with the cathepsin L sequence inserted into the EGFP-encoding vector were cultivated in kanamycin-containing LB medium, and plasmids were harvested from this broth of resistant bacteria. As an additional control of proper vector construction, the purified plasmids were digested with *NheI* and *BamHI* to gain the insert, and additionally with *EcoRI*, which cleaves once within the cathepsin L sequence. Therefore, after digest of cathepsin L-EGFP-containing plasmids, fragments with a length of 4700 bp, 760 bp, and 259 bp were expected. The preparations were analyzed by agarose gel electrophoresis, and three bands of the expected size were observed in the digests of *p*hCL-EGFP (Fig. 2).

**Fig. 3:**
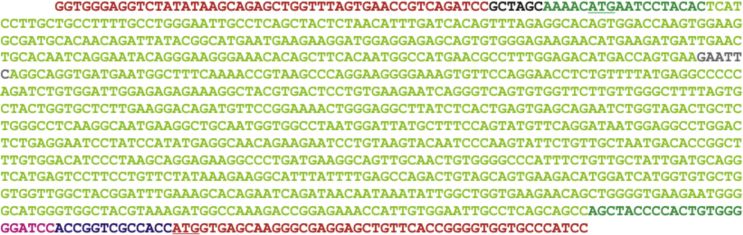
Partial sequence of *p*hCL-EGFP. Nucleotide sequence of insert region of *p*hCL-EGFP as revealed by up- and down-stream sequencing reactions is depicted. The cathepsin L-coding sequence is highlighted in light green, sequences of the surrounding vector are labeled in red, start-codons ATG of cathepsin L and EGFP, respectively, are underlined, primers are marked in dark green, the cleavage site of *NheI* is shown in black, the restriction site of *BamHI* is marked in pink, and the *EcoRI* site in grey, while the spacer sequence between cathepsin L and EGFP is color-coded in dark blue.

**Fig. 4:**
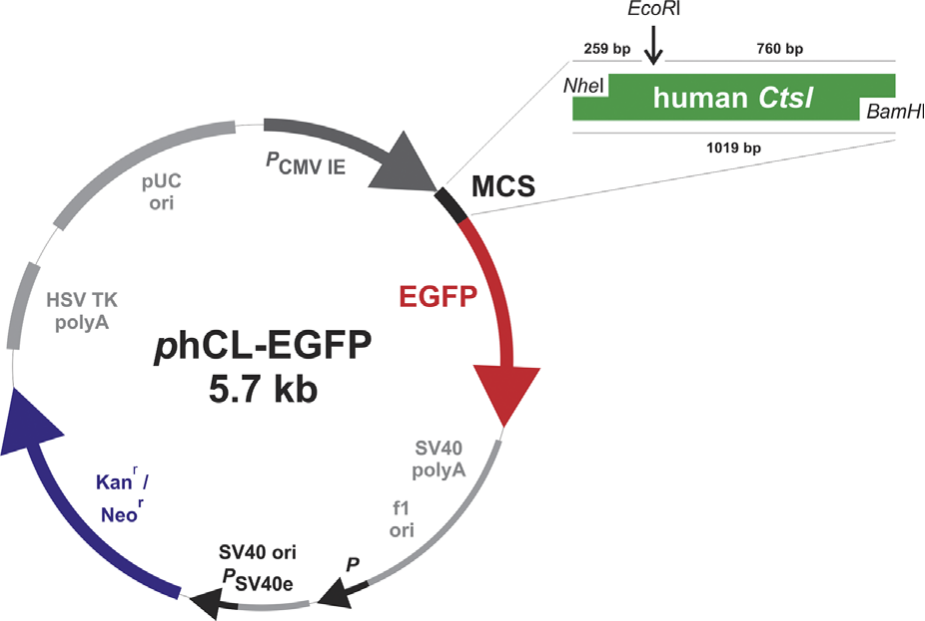
Vector map of *p*hCL-EGFP. Scheme of the 5.7-kb-vector *p*hCL-EGFP coding for full-length cathepsin L-EGFP chimeric protein is depicted. Note that cathepsin L was cloned into the multiple cloning site (MCS) of the *p*EGFP-N1 vector (Clontech) using *NheI* and *BamHI* as indicated. Additionally the restriction site for *EcoRI* within the cDNA sequence that was used for control digests is marked (arrow). Numbers denote the size of full-length cathepsin L-coding cDNA and that of the expected fragments after control digest.

Next, the plasmid *p*hCL-EGFP was sequenced from downstream (starting 5′) and upstream (starting 3′) in the vector (Fig. 3). Alignment of this cDNA sequence with the reported human cathepsin L sequence (preprocathepsin L precursor, *Homo sapiens*, accession number GenBank AAA66974.1) revealed 100% identity, confirming that cathepsin L-coding cDNA was inserted into the *p*EGFP-N1 vector as shown in the vector map of *p*hCL-EGFP (Fig. 4).

## 2.2 Transfection of HCT116 cells with *p*hCL-EGFP

**Fig. 5:**
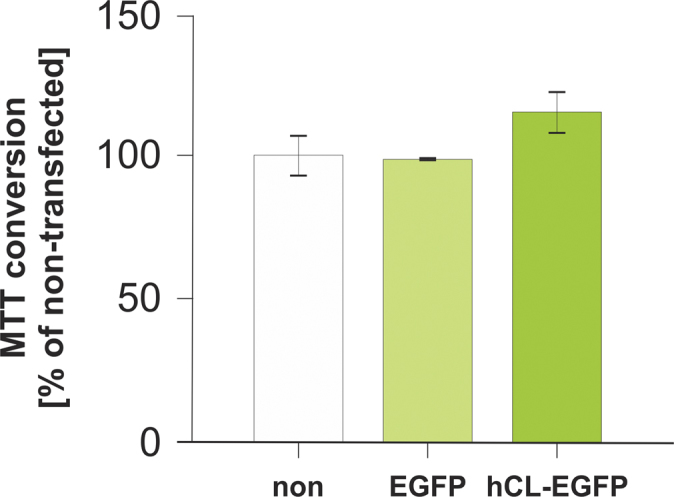
Effects of cathepsin L-EGFP expression on proliferation and metabolic activity of HCT116 cells. Quantitative analyses of cell numbers and metabolic activities by MTT assays were performed with non-transfected HCT116 cells in comparison to cells analyzed 24 h after transfection with *p*EGFP and *p*hCL-EGFP, as indicated. Values are given as means±standard deviations of at least two independent experiments.

Sub-confluent cultures of HCT116 cells were transiently transfected with *p*hCL-EGFP yielding expression of full-length cathepsin L, which was co-localized with endogenous cathepsin L and also reached the nuclei (see Fig. 7D′ in [1]). In addition, MTT assays were performed with non-transfected, *p*EGFP-N1-, and *p*hCL-EGFP-transfected HCT116 cells after 24 h. Data revealed that proliferation rates were not altered upon expression of enhanced GFP alone, while the MTT conversion rates were increased in HCT116 cells expressing hCL-EGFP chimeras (Fig. 5), although this increase did not reach significance over non-transfected controls.

## 2.3 Molecular forms of cathepsin L-EGFP chimeras translated in HCT116 cells

**Fig. 6:**
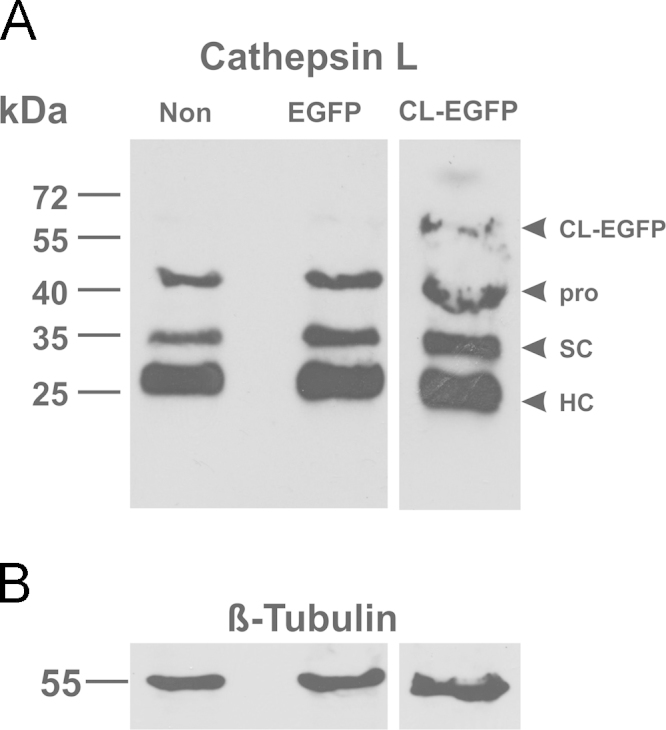
Molecular forms and integrity of transiently expressed cathepsin L-EGFP chimeras in HCT116 cells. Immunoblot analyses were conducted with whole cell lysates of HCT116 cells transiently expressing *p*EGFP-N1 and *p*hCL-EGFP as indicated, in comparison to non-transfected controls. Visualization of bands immunoreactive with cathepsin L-specific antibodies (A) was performed by enhanced chemi-luminescence. Note that the chimeric protein CL-EGFP was detectable at approx. 65 kDa as expected for the intact protein. In addition, all expected molecular forms of cathepsin L were detected with anti-cathepsin L-specific antibodies (pro, SC, and HC). ß-Tubulin was used as a loading control (B). Molecular mass markers are indicated in the left margins.

To identify the molecular forms of the cathepsin L-EGFP chimeras translated in HCT116 cells, immunoblotting was performed with whole cell Iysates of non-transfected cultures and with those expressing *p*EGFP-N1 or *p*hCL-EGFP, respectively. The expected molecular forms of cathepsin L were detected in all samples, i.e. the pro-form as well as the single chain (SC) and heavy chain (HC) of the two-chain form of the protease. Lysates of *p*hCL-EGFP-transfected HCT116 cells featured an additional band at 65 kDa that was recognized by cathepsin L-specific antibodies (Fig. 6), and which was absent from non-transfected cells or those transfected with the empty vector *p*EGFP-N1. The data indicates that the band at 65 kDa is representative of the hCL-EGFP chimeric protein which was stable for 24 h in HCT116 cells because no degradation bands could be observed in immunoblots with cathepsin L-specific antibodies (Fig. 6).

## References

[1] Tamhane T., Illukkumbura R., Lu S., Maelandsmo G.M., Haugen M.H., Brix K. (2015). Nuclear cathepsin L activity is required for cell cycle progression of colorectal carcinoma cells. Biochimie.

[2] Linke M., Herzog V., Brix K. (2002). Trafficking of lysosomal cathepsin B-green fluorescent protein to the surface of thyroid epithelial cells involves the endosomal/lysosomal compartment. J. Cell Sci..

[3] Mayer K., Iolyeva M.E., Meyer-Grahle U., Brix K. (2008). Intestine-specific expression of green fluorescent protein-tagged cathepsin B: proof-of-principle experiments. Biol. Chem..

[4] Tedelind S., Jordans S., Resemann H., Blum G., Bogyo M., Fuhrer D., Brix K. (2011). Cathepsin B trafficking in thyroid carcinoma cells. Thyroid Res..

[5] Brix K., Jordans S. (2005). Watching proteases in action. Nat. Chem. Biol..

[6] Brix K., Dunkhorst A., Mayer K., Jordans S. (2008). Cysteine cathepsins: cellular roadmap to different functions. Biochimie.

[7] Brix K., Scott C.J., Heck M.M.S., Brix K., Stocker W. (2013). Compartmentalization of proteolysis. Proteases: Structure and Function.

[8] Rehders M., Grosshauser B.B., Smarandache A., Sadhukhan A., Mirastschijski U., Kempf J., Dunne M., Slenzka K., Brix K. (2011). Effects of lunar and mars dust simulants on HaCaT keratinocytes and CHO-K1 fibroblasts. Adv. Space Res..

[9] Tamhane T., Arampatzidou M., Gerganova V., Tacke M., Illukkumbura R., Dauth S., Schaschke N., Peters C., Reinheckel T., Brix K. (2014). The activity and localization patterns of cathepsins B and X in cells of the mouse gastrointestinal tract differ along its length. Biol. Chem..

